# AI-Assisted Electrochemical Immunosensing for Matrix-Aware Detection of Aflatoxin M1 and Atrazine in Food Matrices

**DOI:** 10.3390/bios16070352

**Published:** 2026-06-23

**Authors:** Kundan Kumar Mishra, Shanmathi Venkatesan, Sriram Muthukumar, Shalini Prasad

**Affiliations:** 1 Department of Bioengineering, University of Texas at Dallas, Richardson, TX 75080, USA; kundan.mishra@utdallas.edu (K.K.M.);; 2 EnLiSense LLC, 1813 Audubon Pondway, Allen, TX 75013, USA

**Keywords:** Aflatoxin M1, Atrazine, electrochemical immunosensor, electrochemical sensing, machine learning, AI-assisted biosensing

## Abstract

Food contamination by Aflatoxin M1 and Atrazine remains a critical food-safety concern, requiring sensitive detection methods that can operate reliably in complex matrices. Here, we report an AI-assisted antibody-functionalized electrochemical sensing platform for the detection and classification of Aflatoxin M1 and Atrazine across corn, corn flour, and protein matrices. The sensor used analyte-specific antibodies immobilized on an electrochemical electrode surface, where target binding produced measurable changes in the interfacial electrochemical response. Sensor performance was evaluated using cyclic voltammetry, coulometry, and electrochemical impedance spectroscopy (EIS), with EIS providing strong frequency-dependent signatures for concentration-dependent analysis. Spike-and-recovery studies further demonstrated the applicability of the platform in food-matrix conditions. To improve interpretation of complex electrochemical signals, full-spectrum EIS features were integrated with machine learning models for concentration-level classification into low, mid, and high groups. The AI workflow achieved an overall classification accuracy of 93.33%, with 96.67% specificity, 93.44% PPV, 96.66% NPV, and 0.982 AUC for Atrazine, and 96.70% specificity, 93.38% PPV, 96.67% NPV, and 0.987 AUC for Aflatoxin M1. In addition, analyte classification between Aflatoxin M1 and Atrazine reached 97.4% accuracy and 0.994 ROC-AUC. Overall, this work demonstrates a matrix-aware electrochemical immunosensing strategy enhanced by AI-based signal interpretation for food contaminant detection.

## 1. Introduction

Food-safety monitoring increasingly requires analytical platforms that can detect chemically diverse contaminants in complex matrices while remaining rapid, portable, and interpretable. Among these contaminants, Aflatoxin M1 and Atrazine are important because they represent two different classes of food-relevant hazards: mycotoxin residues and agricultural herbicide residues. Aflatoxin M1 (AFM1) is a hydroxylated metabolite of aflatoxin B1 and is commonly associated with food-chain transfer after animals consume contaminated feed; recent reviews continue to highlight the need for sensitive AFM1 detection strategies in milk and food products because of its toxicity, persistence, and regulatory importance [1]. Atrazine, a widely used triazine herbicide, is also a persistent environmental contaminant that can enter agricultural runoff and food-associated water systems, motivating the development of rapid biosensors and electrochemical platforms for field-deployable monitoring [2].

Conventional chromatographic and immunoassay methods provide reliable contaminant quantification, but they often require laboratory infrastructure, trained personnel, and longer turnaround times. In contrast, biosensors offer a route toward rapid, lower-cost, and potentially on-site testing by coupling a selective recognition element with a measurable optical, electrochemical, or mechanical transduction signal. Recent food-safety biosensor reviews emphasize that integrated sensing platforms can improve contaminant screening across the supply chain by enabling faster testing, reduced sample volume, and portable readout formats [3,4]. Within this broader biosensing field, electrochemical sensors are particularly attractive because they are compatible with miniaturized electrodes, low-power instrumentation, multiplexed measurement, and direct conversion of binding events into current, potential, charge, or impedance signals [5,6].

Antibody-based electrochemical immunosensors are well suited for selective food-contaminant detection because antibodies provide high molecular specificity while electrochemical readouts provide sensitivity to interfacial changes at the electrode surface. Early work demonstrated the feasibility of screen-printed electrochemical immunosensors for Aflatoxin M1, including amperometric detection using antibody-modified electrodes [7]. More recent studies have advanced AFM1 sensing using nanobody-functionalized screen-printed electrodes, field-portable electrochemical immunoreceptor designs, dual-recognition electrochemical aptasensor/MIP strategies, and ratiometric electrochemical aptasensors [8,9,10,11]. These studies collectively show that electrochemical formats can support sensitive AFM1 detection, but matrix complexity and sensor-to-sensor variability remain important challenges for practical deployment. Electrochemical sensing has also been widely explored for Atrazine detection. Recent Atrazine biosensor studies include enzyme-based electrochemical sensors, label-free electrochemical immunosensors, green-synthesized nanoparticle-based electrochemical approaches, and biomimetic/molecularly imprinted nanosensors for field or environmental monitoring [12,13,14,15]. Reviews of atrazine and pesticide electrochemical biosensors further emphasize that immunosensors, aptasensors, enzyme-based sensors, and molecularly imprinted platforms can improve selectivity and portability compared with conventional methods [16]. However, many reported systems focus on one analyte, one matrix, or one primary electrochemical response mode, leaving a need for sensing workflows that can evaluate multiple contaminants across different food-relevant backgrounds.

A central challenge in electrochemical food sensing is the matrix effect. Food matrices can contain proteins, carbohydrates, lipids, salts, suspended solids, and redox-active interferents that alter background conductivity, electrode fouling, nonspecific adsorption, and double-layer structure. These effects can shift the electrochemical baseline and distort the relationship between analyte concentration and signal. This is especially relevant for impedance-based sensors, where the response depends on frequency-dependent charge transfer, interfacial capacitance, solution resistance, and surface binding. Reviews of electrochemical immunosensors and impedance-based sensing emphasize that matrix composition and interfacial architecture can strongly influence analytical performance and must be considered during calibration and validation [17,18]. Therefore, a matrix-aware sensing strategy should not only demonstrate calibration in controlled conditions but also evaluate spike recovery, cross-reactivity, and performance across multiple matrices.

Electrochemical impedance spectroscopy (EIS) is particularly powerful for this purpose because it produces a frequency-resolved fingerprint rather than a single current or potential value. Binding of an analyte to an antibody-functionalized electrode can change the impedance magnitude, real impedance, imaginary impedance, and phase angle across different frequency regions. Low-frequency features may reflect charge-transfer resistance and double-layer effects, while mid- and high-frequency features may capture solution resistance, capacitive relaxation, or electrode-surface heterogeneity. Because these responses are distributed across the spectrum, full-spectrum EIS can contain more information than a single-frequency readout. This has motivated recent work combining EIS and advanced data analysis for biosensing, including portable EIS immunosensors coupled with machine learning for quantitative prediction when impedance responses are not strictly linear with concentration [19]. Machine learning offers an additional layer of value because it can extract nonlinear relationships from high-dimensional electrochemical data and convert complex sensor outputs into classification or prediction decisions. Recent reviews of ML-enhanced electrochemical sensors and ML-powered biosensors for food safety describe how algorithms can support feature extraction, classification, predictive modeling, and real-time decision-making for contaminants such as pesticides, mycotoxins, heavy metals, microorganisms, and adulterants [20,21,22,23]. AI-assisted biosensing has also been highlighted as an emerging route for improving food-safety analysis by enabling data-driven interpretation of signals generated from complex matrices [24,25]. However, strong performance under random cross-validation alone is not sufficient; robust deployment also requires evaluating whether models generalize across unseen matrices and sensor chips [26,27].

**Figure 1:**
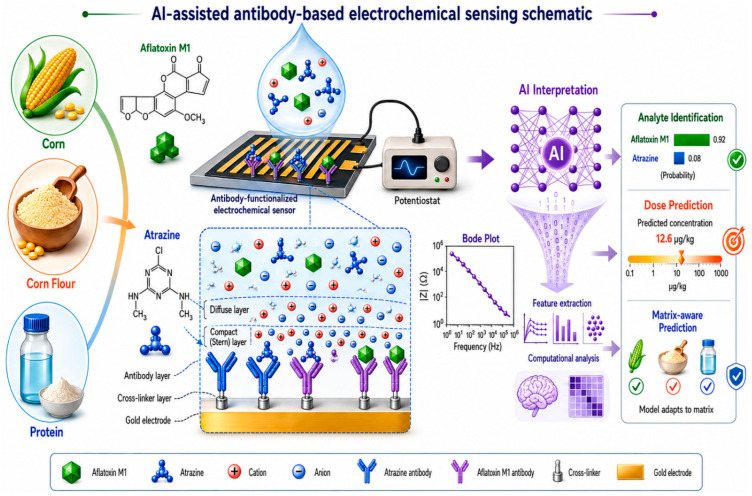
Schematic overview of the antibody-functionalized electrochemical sensor for detecting Aflatoxin M1 and Atrazine in corn, corn flour, and protein matrices. Target binding at the antibody-modified electrode generates impedance-based electrochemical fingerprints, which are processed by AI for analyte identification, dose prediction, and matrix-aware classification.

In this work, we present an AI-assisted antibody-functionalized electrochemical sensing platform for the matrix-aware detection of Aflatoxin M1 and Atrazine across corn, corn flour, and protein matrices (schematic shown in Figure 1). The platform integrates antibody-based recognition with cyclic voltammetry, coulometry, and EIS to capture complementary electrochemical signatures. Spike-recovery and cross-reactivity studies are used to evaluate practical quantification and selectivity, while full-spectrum EIS features are used to train machine learning models for analyte classification and concentration-level grouping into low, mid, and high categories. By combining antibody-based electrochemical sensing with AI-enabled EIS interpretation, this study addresses two key needs in food-safety biosensing: selective detection of chemically distinct contaminants and data-driven mitigation of matrix-dependent signal complexity.

## 2. Materials and Methods

### 2.1. Materials and Reagents

Monoclonal antibodies specific to Aflatoxin M1 and Atrazine were used as the biorecognition elements for sensor functionalization, procured from Invitrogen (Gaithersburg, MD, USA). The crosslinker DTSSP ($3,3^{\prime }$-dithiobis(sulfosuccinimidyl propionate)) was obtained from Thermo Fisher Scientific Inc. (Waltham, MA, USA) and used to immobilize the antibodies onto the electrode surface. Standard solutions of Aflatoxin M1 and Atrazine were prepared at the required concentrations for calibration, spike-recovery, and cross-reactivity experiments. Corn, corn flour, and protein (purchased from local supermarket) matrices were used as representative food matrices for evaluating matrix-dependent sensor performance. All samples and reagents were divided into smaller aliquots and stored at −20 °C until use. Before each experiment, the aliquots were thawed to room temperature and centrifuged when required to remove suspended particulates. All chemicals and reagents used in this study were of analytical grade, and no additional purification was performed before use.

### 2.2. Instrumentation

Electrochemical measurements were performed using a Gamry Reference 600 potentiostat (Gamry Instruments, Warminster, PA, USA) connected to the antibody-functionalized PCB-based sensing platform. The sensor was operated in a three-electrode configuration consisting of a gold working electrode, carbon counter electrode, and Ag/AgCl reference electrode. Electrochemical impedance spectroscopy (EIS), cyclic voltammetry (CV), and coulometry measurements were carried out using the same potentiostat setup at room temperature. EIS measurements were performed under non-Faradaic conditions without the addition of an external redox probe. Potentiostatic EIS was recorded at a DC potential of 0 V using a 10 mV rms AC perturbation over a frequency range of 100 kHz to 10 Hz with 10 points per decade. CV measurements were performed from 0 V to +0.4 V, followed by a reverse scan to −0.6 V and then back to 0 V, using a scan rate of 50 mV/s and a step size of 2 mV. Coulometry measurements were performed using a 0 V pre-step for 10 s, followed by −0.3 V and +0.3 V potential steps of 0.5 s each, with a sampling period of 0.01 s. The recorded electrochemical data were exported for further processing, calibration analysis, recovery evaluation, and AI-based classification.

### 2.3. Sensor Fabrication

The electrochemical sensor was fabricated on a laboratory-designed printed circuit board (PCB) platform using a three-electrode configuration. The sensing platform consisted of a gold working electrode, a carbon counter electrode, and an Ag/AgCl reference electrode. The electrode array was patterned on a PCB-FR4 substrate with an electroless nickel immersion gold (ENIG) finish on a copper-clad board. Each sensing region was designed to provide a stable electrochemical interface for antibody immobilization and target detection. The integrated Ag/AgCl reference electrode helped maintain a stable reference potential during electrochemical measurements, while the gold working electrode provided a suitable surface for DTSSP-mediated antibody functionalization. This PCB-based electrode design enabled reproducible electrochemical measurements across different chips and was used for EIS, CV, and coulometry analysis of Aflatoxin M1 and Atrazine in food matrices.

### 2.4. Modification of the Sensing Platform

The electrode surface was first cleaned with phosphate-buffered saline (PBS) to remove loosely bound contaminants and prepare the sensing area for functionalization. The cleaned electrodes were then modified using DTSSP ($3,3^{\prime }$-dithiobis(sulfosuccinimidyl propionate)) as the crosslinker to enable antibody immobilization on the electrode surface. A mixture of DTSSP (10 mM) and analyte-specific antibodies was applied onto the sensor surface and incubated at 4 °C for 30 min. Separate antibody-functionalized sensing regions were prepared for Aflatoxin M1 and Atrazine detection. After incubation, the sensor was rinsed with PBS to remove unbound crosslinker and excess antibody. For sample analysis, 5 µL of the prepared matrix-spiked sample was added onto the modified sensing surface and incubated for 5 min to allow antibody–target binding. The tested samples included corn, corn flour, and protein matrices containing different concentrations of Aflatoxin M1 or Atrazine. After the binding step, electrochemical measurements were performed using EIS, CV, and coulometry to evaluate the target-induced changes at the antibody-functionalized electrode interface. The resulting electrochemical signals were then used for calibration, spike-recovery analysis, cross-reactivity evaluation, and AI-based classification.

### 2.5. Data Processing and Cleaning

Raw electrochemical data from EIS, CV, and coulometry were organized according to analyte, matrix, dose level, repeat, chip, and measurement type. Dose labels were standardized across all files, with the blank/control condition assigned as D0. For Aflatoxin M1, D0–D5 corresponded to 0, 0.01, 0.055, 0.303, 1.664, and 9.151 ng/mL. For Atrazine, D0–D5 corresponded to 0, 0.1, 0.4, 1.6, 6.4, and 25.6 ng/mL.

For EIS analysis, frequency, ∣Z∣, $Z^{\prime }$, $Z^{\prime \prime }$, and phase angle were extracted and aligned across samples. The EIS spectra were processed as full-spectrum impedance fingerprints rather than being reduced to equivalent circuit model parameters. Equivalent circuit model fitting was not used as the primary analysis method because the measurements were performed under non-Faradaic conditions and the spectra reflected composite interfacial responses. For CV analysis, potential and current were standardized, and current was converted to microampere for plotting. For coulometry, time, current, and charge were extracted and converted to suitable units for visualization. Replicate data were retained during processing, and final plots were generated using averaged responses within each analyte–matrix–dose group.

For calibration and response analysis, chip-level means were first calculated to avoid over-weighting technical repeats. Final values were reported as mean response across different chips, with error bars shown as standard error of the mean (SEM). Percentage change in impedance was calculated relative to the blank response using:

$$\%\Delta |Z|=\frac{|Z{|}_{\mathrm{blank}}-|Z{|}_{\mathrm{sample}}}{|Z{|}_{\mathrm{blank}}}\times 100$$

For matrix-effect reduction, each sample was normalized against its corresponding matrix-specific blank response before downstream calibration and AI analysis. This blank-normalization step was used to reduce baseline differences caused by matrix composition, chip-to-chip variation, and nonspecific background impedance before evaluating concentration-dependent trends. The cleaned and processed datasets were used for electrochemical plotting, calibration, recovery analysis, cross-reactivity evaluation, and machine learning feature extraction.

## 3. Results and Discussion

### 3.1. Electrochemical Fingerprints Reveal Dose-Dependent Antibody–Target Recognition

**Figure 2:**
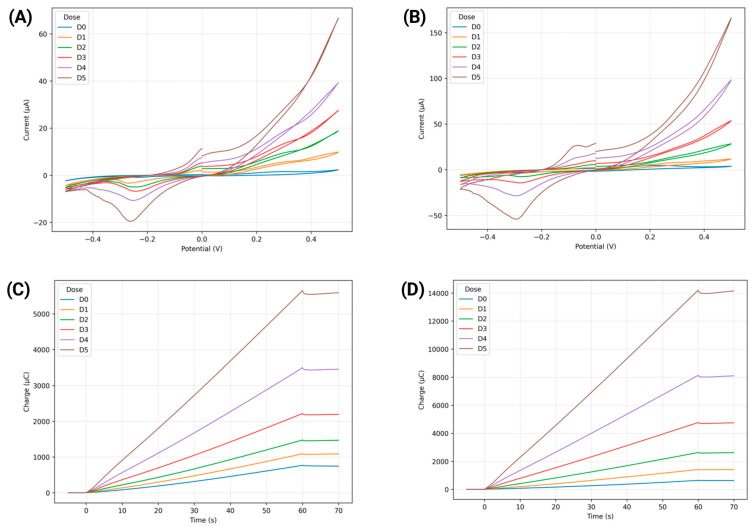
(**A**) CV response of Aflatoxin M1 across D0–D5 concentrations. (**B**) CV response of Atrazine across D0–D5 concentrations. The CV curves show concentration-dependent current modulation, indicating that antibody–target binding changes the interfacial electrochemical environment. (**C**) Coulometric response of Aflatoxin M1 showing time-dependent charge/current accumulation with increasing concentration. (**D**) Coulometric response of Atrazine showing dose-dependent separation across the tested concentration range. For Aflatoxin M1, D0–D5 correspond to 0, 0.01, 0.055, 0.303, 1.664, and 9.151 ng/mL. For Atrazine, D0–D5 correspond to 0 ng/mL, 0.1 ng/mL, 0.4 ng/mL, 1.6 ng/mL, 6.4 ng/mL, and 25.6 ng/mL.

The antibody-functionalized electrode generated distinct electrochemical responses after exposure to increasing concentrations of Aflatoxin M1 and Atrazine, confirming that target binding produced measurable changes at the electrode–solution interface. In electrochemical immunosensors, the antibody layer acts as the biological recognition element, while antigen binding is transduced into an electrical signal through changes in interfacial charge transfer, surface accessibility, double-layer behavior, and local electron-transfer kinetics. This mechanism is consistent with established electrochemical immunosensor principles, where antibody–antigen interactions at the electrode surface modulate voltammetric or amperometric signals [28,29]. Although Aflatoxin M1 and Atrazine are small molecules, their binding occurs within a much larger immobilized antibody layer at the electrode interface. Therefore, the measured signal is not governed only by the molecular size of the analyte, but also by the cumulative changes produced in the antibody-functionalized interfacial film. Target binding can alter antibody orientation, hydration structure, surface charge distribution, local dielectric properties, and ion accessibility near the electrode surface, producing amplified electrochemical changes. In Figure 2A,B, the cyclic voltammetry responses show concentration-dependent current modulation for both analytes. CV measurements were performed from 0 V to +0.4 V, followed by a reverse scan to −0.6 V and then back to 0 V, using a scan rate of 50 mV/s and a step size of 2 mV. For Aflatoxin M1, the current response progressively changes from the blank/low-dose condition toward higher dose levels, indicating that Aflatoxin M1 binding alters the interfacial electrochemical environment. The stronger response at higher concentrations suggests increased surface occupation by the antibody–antigen complex, which can modify ionic accessibility, interfacial polarization, and capacitive charging at the electrode surface [30,31,32,33,34]. For Atrazine, the CV curves also demonstrate a dose-dependent response, supporting successful recognition by the anti-Atrazine antibody layer. The change in current with increasing concentration suggests that Atrazine binding modifies the surface charge environment and electron-transfer behavior of the electrode [35,36]. Because no external redox probe was used, the CV response is interpreted primarily as an interfacial/capacitive response rather than direct redox conversion of Aflatoxin M1 or Atrazine. The peak observed at negative potentials is likely associated with cathodic interfacial charging, surface polarization, and surface-associated electrochemical processes at the DTSSP/antibody-modified electrode surface. This feature may also include contributions from trace electrolyte or matrix-associated species; therefore, it was not assigned to a specific redox couple. The CV curves were plotted directly from the exported forward and reverse scan traces without artificially forcing closure at 0 V. Therefore, slight differences between the initial and final currents near 0 V may arise from capacitive charging, surface polarization, adsorption-related interfacial changes, and matrix-associated background effects during the scan. Accordingly, the CV profiles were interpreted as concentration-dependent interfacial current modulations rather than classical closed reversible redox loops.

The coulometric responses in Figure 2C,D provide additional evidence of concentration-dependent electrochemical behavior. Unlike CV, which captures current response during a potential sweep, coulometry evaluates the time-dependent current or accumulated charge under a fixed potential condition. The observed increase or separation in cumulative charge across dose levels indicates that target concentration influences the total charge-transfer process over time. Similar to the CV response, the coulometric signal reflects binding-induced modulation of interfacial charge accumulation and ion transport within the antibody-functionalized layer, rather than direct electrochemical oxidation or reduction of the target analytes. This is important because charge integration can reduce the impact of short-term current fluctuations and provide a more stable representation of the binding-induced electrochemical response. Together, the CV and coulometric results show that both Aflatoxin M1 and Atrazine produce measurable, dose-dependent electrochemical signatures on the antibody-functionalized sensor. The CV curves confirm potential-dependent signal modulation, while the coulometric curves confirm a time-integrated charge response. These findings support the use of the electrochemical outputs as quantitative features for later EIS calibration and AI-based concentration classification. Therefore, Figure 2 establishes the first experimental validation that antibody–target binding produces analyte-responsive electrochemical signals across the tested concentration range.

### 3.2. Frequency-Resolved Impedance Signatures Enable Quantitative Detection of Aflatoxin M1 and Atrazine

Electrochemical impedance spectroscopy provides the most informative signal for quantitative analysis because it captures how antibody–target binding changes the interfacial resistance, capacitance, and charge-transfer behavior of the electrode surface. In this study, EIS was performed under non-Faradaic conditions without the addition of an external redox probe. Therefore, the impedance response was not interpreted as redox-probe-mediated electron transfer, but rather as target-induced modulation of the antibody-functionalized electrode/electrolyte interface. In antibody-functionalized electrochemical sensors, the formation of an antibody–antigen complex modifies the electrical double layer and restricts or alters ion/electron transport near the electrode [37,38]. Binding Aflatoxin M1 or Atrazine to the immobilized antibody layer can change the local dielectric environment, hydration structure, surface charge distribution, steric accessibility, and ionic organization near the electrode surface. These interfacial changes influence double-layer capacitance, surface polarization, and ion redistribution, which are especially prominent in the low-frequency region of the EIS spectrum. This makes EIS especially useful for label-free or minimally labeled biosensing, where target recognition is translated into measurable impedance changes. Impedimetric biosensors have been widely used in food-safety detection because they can sensitively monitor surface-binding events through frequency-dependent impedance responses [39].

**Figure 3:**
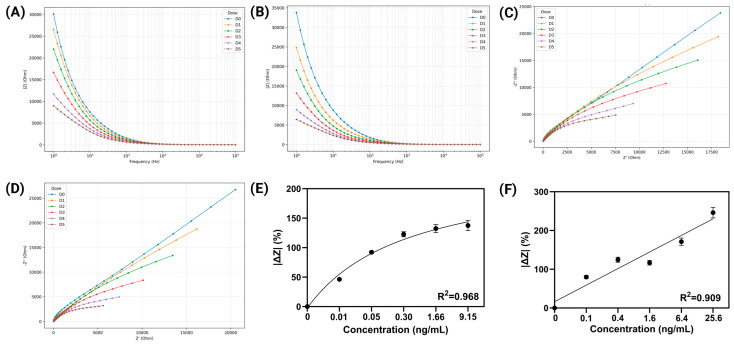
(**A**) Bode magnitude response for Aflatoxin M1 across D0–D5 concentrations, showing concentration-dependent impedance modulation. (**B**) Bode magnitude response for Atrazine across D0–D5 concentrations. The strongest impedance response is observed in the low-frequency region, where interfacial charge-transfer and double-layer effects dominate the signal. (**C**) Concentration-dependent impedance response for Aflatoxin M1 extracted from the EIS spectra. (**D**) Concentration-dependent impedance response for Atrazine extracted from the EIS spectra. (**E**) Calibration curve for Aflatoxin M1 generated from (|Z|) values extracted at approximately 198 Hz, showing a strong dose–response relationship with R^2^ = 0.968. (**F**) Calibration curve for Atrazine showing concentration-dependent response with R^2^ = 0.909. For Aflatoxin M1, D0–D5 represent 0 ng/mL, 0.01 ng/mL, 0.055 ng/mL, 0.303 ng/mL, 1.664 ng/mL, and 9.151 ng/mL; for Atrazine, D0–D5 represent 0 ng/mL, 0.1 ng/mL, 0.4 ng/mL, 1.6 ng/mL, 6.4 ng/mL, and 25.6 ng/mL.

In Figure 3A,B, the frequency-dependent Zmod responses show the typical EIS behavior expected from an interfacial biosensor: the impedance is highest at a low frequency and gradually decreases as the frequency increases. This trend occurs because low-frequency measurements allow more time for interfacial polarization, double-layer charging, and antibody–analyte binding effects to influence the signal. At low frequencies, ions can redistribute near the modified electrode surface during the applied AC perturbation, making the impedance response highly sensitive to changes in the antibody layer after target binding. At higher frequencies, the response becomes more dominated by bulk solution resistance and rapid capacitive behavior, reducing the visible difference between concentration groups. Therefore, the low-to-mid-frequency region is particularly important for extracting sensing information from the antibody-functionalized interface. For Aflatoxin M1, the Zmod response in Figure 3A shows clear concentration-dependent modulation across the frequency range, with larger separation at lower frequencies. This suggests that Aflatoxin M1 binding produces a strong interfacial impedance change, likely due to increased surface loading and altered charge-transfer resistance at the antibody-modified electrode [40]. For Atrazine, the Zmod response in Figure 3B also follows a concentration-dependent impedance trend. Although the overall shape is similar to Aflatoxin M1, the magnitude and separation pattern differ, indicating that the anti-Atrazine antibody interface produces a distinct impedance signature. This difference is important because it supports the later AI-based classification of the two analytes [41,42,43]. The Nyquist responses in Figure 3C,D further confirm the concentration-dependent impedance behavior of the antibody-functionalized sensor. In these plots, the real impedance component $\left(Z^{\prime }\right)$ is plotted against the negative imaginary impedance component (−Z″), providing insight into charge-transfer and interfacial processes at the electrode surface. For both Aflatoxin M1 and Atrazine, the Nyquist curves show dose-dependent changes across D0–D5, indicating that antibody–target binding progressively modifies the electrode interface. Because the system was operated without an external redox probe, the Nyquist response is interpreted as a composite non-Faradaic interfacial impedance response rather than a classical redox-mediated charge-transfer process. The variation in curve shape and impedance magnitude suggests changes in charge-transfer resistance and double-layer behavior as more target molecules bind to the antibody layer. This response is important because Nyquist analysis helps visualize how the interfacial electrochemical properties evolve with concentration, supporting the use of EIS as a quantitative sensing method. Together with the Bode magnitude plots, the Nyquist responses demonstrate that the sensor signal is both frequency-dependent and concentration-dependent, enabling conversion of the raw impedance spectra into calibration curves for Aflatoxin M1 and Atrazine detection. The calibration plots in Figure 3E,F further demonstrate the analytical potential of the sensor. These calibration curves were generated using impedance magnitude (|Z|) values extracted from the EIS spectra at approximately 198 Hz, a representative frequency within the measured non-Faradaic EIS range where clear concentration-dependent separation was observed. For Aflatoxin M1, the calibration curve shows a strong concentration-dependent relationship with an R^2^ of 0.968, indicating good agreement between the concentration and impedance response. This suggests that the Aflatoxin M1 antibody interface provides a stable and reproducible impedance response across the tested concentration range. For Atrazine, the calibration curve shows an R^2^ of 0.909, which still supports quantitative detection, although the fit is weaker than that for Aflatoxin M1. The lower (R^2^) observed for Atrazine compared with Aflatoxin M1 may reflect analyte- and matrix-dependent variability in the impedance response rather than a specific limitation of the sensing surface. Atrazine may be more sensitive to matrix-associated effects because its physicochemical properties can influence its distribution, accessibility, and interaction with the electrode–solution interface in complex food matrices. Matrix components such as starch, proteins, and suspended residues may alter local ion transport, dielectric behavior, and nonspecific background impedance, leading to greater variability in the Atrazine calibration response. In contrast, Aflatoxin M1 produced a more reproducible concentration-dependent impedance trend under the tested conditions. Overall, the EIS results show that both Aflatoxin M1 and Atrazine generate concentration-dependent impedance signatures on the antibody-functionalized sensor. The stronger response at low and mid-frequencies confirms that target binding mainly affects the electrode interface rather than only the bulk solution. These results support a non-Faradaic sensing mechanism in which antibody–target recognition modulates interfacial capacitance, surface polarization, and ionic redistribution at the electrode/electrolyte interface. The calibration performance, especially the strong Aflatoxin M1 response, supports the use of Zmod-derived features for quantitative analysis. These results also justify the use of full-spectrum EIS features in the AI workflow, because the analyte-specific information is distributed across the frequency rather than limited to a single point. Thus, Figure 3 establishes EIS as the core analytical readout for both calibration and downstream machine learning classification in this sensing platform.

### 3.3. Spike-Recovery and Cross-Reactivity Confirm Matrix Compatibility and Sensor Selectivity

**Figure 4:**
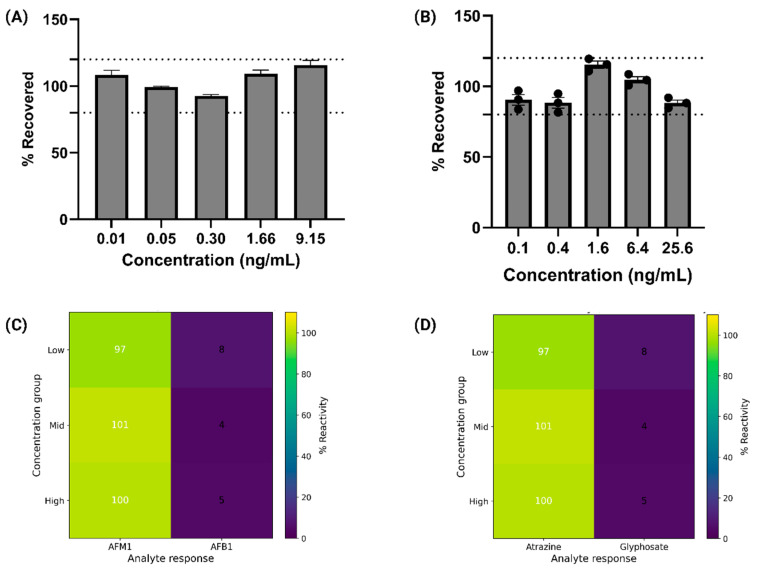
(**A**) Spike-recovery performance for Aflatoxin M1 across 0.01, 0.055, 0.303, 1.664, and 9.151 ng/mL, showing recovery values close to the expected 100% response and largely within the 80–120% (presented in dashed line) acceptable recovery window. (**B**) Spike-recovery performance for Atrazine across 0.1, 0.4, 1.6, 6.4, and 25.6 ng/mL, demonstrating concentration-dependent recovery within the acceptable analytical range. Dashed horizontal lines indicate the recovery acceptance limits, and error bars represent replicate variation. (**C**) Cross-reactivity matrix for the Aflatoxin M1 sensor, showing strong target response to Aflatoxin M1 across low-, mid-, and high-concentration groups, with minimal response to the non-target interferent Aflatoxin B1. (**D**) Cross-reactivity matrix for the Atrazine sensor, showing high target response to Atrazine and low cross-reactive response to glyphosate.

Spike-and-recovery analysis was performed to evaluate whether the antibody-functionalized electrochemical sensor could accurately quantify Aflatoxin M1 and Atrazine in matrix-spiked samples. This validation step is important because complex food matrices can introduce nonspecific adsorption, ionic background changes, and surface fouling, which may affect the electrochemical response. Therefore, recovery close to 100% indicates that the sensor response remains reliable even in the presence of matrix components. For Aflatoxin M1, the recovery response in Figure 4A remained within the acceptable analytical range across the tested concentrations of 0.01, 0.055, 0.303, 1.664, and 9.151 ng/mL. The recovery values were centered near 100%, with only small concentration-dependent variation. This suggests that the Aflatoxin M1 antibody interface maintained stable recognition and electrochemical response across low, mid-, and high concentration levels. The slightly lower recovery at the mid-concentration may reflect minor matrix interference or local variation in antibody–antigen binding, but the overall response confirms reliable quantification. For Atrazine, the spike-recovery response in Figure 4B also showed strong matrix compatibility across 0.1, 0.4, 1.6, 6.4, and 25.6 ng/mL. Most recovery values were close to the 100% reference level and remained within the 80–120% recovery window. The highest response was observed near the mid-concentration, while the highest concentration showed slightly lower recovery. This may indicate mild signal saturation or matrix-dependent suppression at higher Atrazine loading, but the overall recovery profile supports quantitative detection across the tested range [44,45].

Sensor selectivity was further evaluated using cross-reactivity analysis. In Figure 4C, the Aflatoxin M1 sensor showed a strong target response for Aflatoxin M1, with reactivity values of approximately 97%, 101%, and 100% across low-, mid-, and high-concentration groups. In contrast, the structurally related interferent Aflatoxin B1 produced much lower responses of approximately 8%, 4%, and 5%, respectively. This large difference confirms that the antibody-functionalized interface preferentially recognizes Aflatoxin M1 and shows minimal cross-reactivity toward Aflatoxin B1. Similarly, Figure 4D demonstrates high selectivity of the Atrazine sensor. The target Atrazine response remained high across low, mid, and high groups, with reactivity near 97–101%, while the non-target herbicide glyphosate produced only weak responses of approximately 4–8%. This indicates that the anti-Atrazine antibody layer provides selective recognition of Atrazine with limited interference from glyphosate [46]. Overall, Figure 4 confirms two important analytical strengths of the sensing platform. First, the spike-recovery results show that both Aflatoxin M1 and Atrazine can be quantified in matrix-spiked conditions with recoveries close to the expected range. Second, the cross-reactivity results demonstrate strong target specificity, with a high response to the intended analyte and minimal response to non-target compounds. Together, these results support the practical applicability of the antibody-functionalized electrochemical sensor for selective food-contaminant detection in complex matrices.

### 3.4. AI-Enabled EIS Fingerprinting Enables Analyte and Concentration-Level Classification

**Figure 5:**
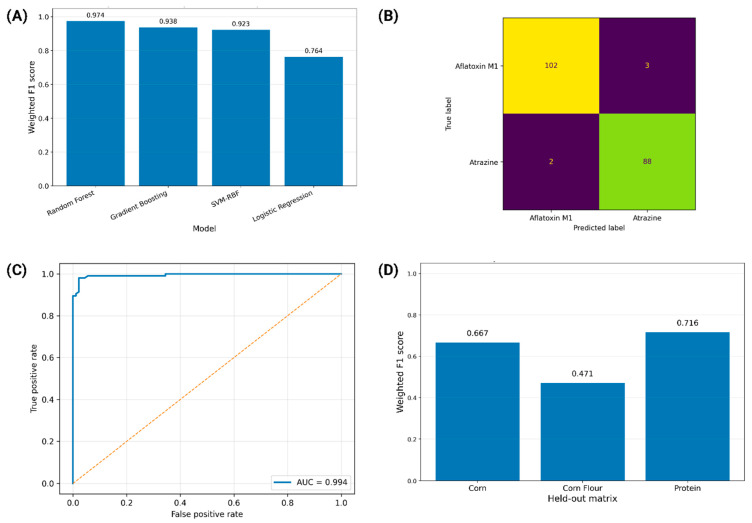
(**A**) Model comparison for analyte classification using full-spectrum EIS features, showing that Random Forest achieved the highest weighted F1 score. (**B**) Random Forest confusion matrix showing correct classification of 102/105 Aflatoxin M1 samples and 88/90 Atrazine samples, corresponding to an overall analyte-classification accuracy of 97.4%. (**C**) ROC curve for Aflatoxin M1 versus Atrazine classification, showing excellent discrimination with AUC = 0.994. (**D**) Leave-one-matrix-out validation showing weighted F1 scores of 0.667 for corn, 0.471 for corn flour, and 0.716 for protein, demonstrating that the AI model can classify analytes using EIS fingerprints but still experiences matrix-dependent variation. For concentration-level classification, D0–D1 were defined as Low, D2–D3 as Mid, and D4–D5 as High.

Full-spectrum EIS features were used to evaluate whether the antibody-functionalized sensor could distinguish Aflatoxin M1 from Atrazine and further classify the samples into concentration-risk groups. Instead of relying on a single impedance value, the AI workflow used the frequency-dependent electrochemical fingerprint generated from ∣Z∣, $Z^{\prime }$, $Z^{\prime \prime }$, and phase features. This is important because antibody–target binding can influence different regions of the impedance spectrum through changes in interfacial charge transfer, double-layer behavior, and matrix-dependent background effects. Therefore, the machine learning model was trained to extract distributed spectral patterns that may not be obvious from individual EIS plots alone. In Figure 5A, the Random Forest confusion matrix shows strong analyte discrimination between Aflatoxin M1 and Atrazine. The model correctly classified 102 out of 105 Aflatoxin M1 samples and 88 out of 90 Atrazine samples, giving an overall analyte-classification accuracy of 97.4%. Only five samples were misclassified, indicating that the two antibody–analyte systems produced distinguishable EIS fingerprints. This result suggests that the sensing platform does not merely detect a generic impedance change, but instead generates analyte-specific spectral signatures that can be learned by the AI model.

The ROC curve in Figure 5B further supports the strength of the classification model. The model achieved an AUC of 0.994, showing excellent separation between Aflatoxin M1 and Atrazine across classification thresholds. This high AUC indicates that the model maintained strong discriminatory power and was not dependent on a single arbitrary decision cutoff. In practical food-safety screening, this is useful because classification thresholds can be adjusted depending on whether the priority is minimizing false positives or false negatives. In Figure 5C, the feature-importance analysis identifies the EIS features that contributed most strongly to analyte classification. The highest-ranked features were mainly derived from phase and real-impedance components at selected frequencies, indicating that analyte discrimination is not controlled only by impedance magnitude. This supports the use of full-spectrum EIS rather than a single-frequency readout. The importance of phase-related features also suggests that antibody–target binding may alter capacitive and interfacial relaxation behavior, which can be captured more effectively through frequency-resolved EIS analysis. The cross-matrix validation in Figure 5D evaluates whether the trained model can generalize to an unseen matrix. In this test, one matrix was removed during training and used only for testing. The model achieved weighted F1 scores of 0.667 for corn, 0.471 for corn flour, and 0.716 for protein. These results show that the AI model can classify analyte-specific electrochemical fingerprints across mixed matrices while also highlighting that matrix composition contributes meaningful background information to the full-spectrum EIS response. The weaker performance for corn flour indicates that this matrix introduces stronger background effects, possibly due to differences in particulate content, viscosity, nonspecific adsorption, or ionic environment. Importantly, this result does not indicate failure of the sensing platform in new matrices; rather, it demonstrates the value of matrix-aware calibration for improving model transferability in complex food samples. Therefore, matrix-aware calibration or inclusion of representative matrix samples during training will be important for future deployment. This finding is a useful outcome of the AI workflow because it identifies matrix-dependent variability that may not be evident from single-matrix calibration alone and supports the development of more robust deployment strategies. In addition to analyte classification, the AI workflow was also used for concentration-level classification into low, mid, and high groups. The dose groups were defined as Low = D0–D1, Mid = D2–D3, and High = D4–D5. The results are summarized in Table 1. Using full-spectrum EIS features, the model achieved an overall concentration-level classification accuracy of 93.33%, with high specificity, PPV, NPV, and AUC for both analytes.

Table 1 shows that the AI model classified concentration levels with strong diagnostic performance for both targets. For Atrazine, the model achieved 96.67% specificity, 93.44% PPV, 96.66% NPV, and an AUC of 0.982. For Aflatoxin M1, the model achieved 96.70% specificity, 93.38% PPV, 96.67% NPV, and an AUC of 0.987. The similar performance across both analytes indicates that the EIS feature space contains sufficient information not only for analyte identification but also for concentration-level grouping. Overall, Figure 5 and Table 1 demonstrate that AI substantially improves interpretation of the electrochemical dataset. The model accurately separated Aflatoxin M1 from Atrazine, achieved high concentration-level classification performance, and identified informative EIS features for decision-making. However, the cross-matrix validation also shows that matrix effects remain an important limitation. Thus, the AI workflow provides both a strong classification tool and a diagnostic framework for identifying where additional matrix normalization or expanded training data are needed.

## 4. Conclusions

This study demonstrates an AI-assisted antibody-functionalized electrochemical sensing platform for matrix-aware detection of Aflatoxin M1 and Atrazine in corn, corn flour, and protein matrices. The sensor produced concentration-dependent responses using CV, coulometry, and EIS, confirming that antibody–target binding generated measurable interfacial electrochemical changes. EIS provided the most informative full-spectrum signal for both calibration and AI-based interpretation. Spike-recovery results showed that both analytes could be quantified with recoveries largely within the acceptable analytical range, while cross-reactivity testing confirmed strong selectivity, with a high target response and minimal interference from Aflatoxin B1 and glyphosate. The AI workflow enabled low/mid/high concentration classification with 93.33% overall accuracy, achieving AUC values of 0.982 for Atrazine and 0.987 for Aflatoxin M1. Full-spectrum EIS features also enabled analyte classification between Aflatoxin M1 and Atrazine with 97.4% accuracy and 0.994 ROC-AUC. Although cross-matrix validation showed that matrix effects still influenced model generalization, the results establish this platform as a promising step toward portable, selective, and AI-enhanced electrochemical food-contaminant detection.

## Figures and Tables

**Table 1:** AI-based low/mid/high concentration-level classification performance using full-spectrum EIS features.

Analysis Group	Samples	Accuracy (%)	Specificity (%)	PPV (%)	NPV (%)	AUC
Overall	195	93.33	96.68	93.41	96.67	0.985
Atrazine	90	93.33	96.67	93.44	96.66	0.982
Aflatoxin M1	105	93.33	96.70	93.38	96.67	0.987

## Data Availability

The data that support the findings of this study are available on request from the corresponding author. The data are not publicly available due to privacy or ethical restrictions.
